# Expression and role of anion exchanger 1 in esophageal squamous cell carcinoma

**DOI:** 10.18632/oncotarget.14900

**Published:** 2017-01-30

**Authors:** Atsushi Shiozaki, Michihiro Kudou, Daisuke Ichikawa, Hiroki Shimizu, Tomohiro Arita, Toshiyuki Kosuga, Hirotaka Konishi, Shuhei Komatsu, Hitoshi Fujiwara, Kazuma Okamoto, Mitsuo Kishimoto, Yoshinori Marunaka, Eigo Otsuji

**Affiliations:** ^1^ Division of Digestive Surgery, Department of Surgery, Kyoto Prefectural University of Medicine, Kyoto, 602-8566, Japan; ^2^ Department of Pathology, Kyoto Prefectural University of Medicine, Kyoto, 602-8566, Japan; ^3^ Department of Molecular Cell Physiology and Bio-Ionomics, Graduate School of Medical Science, Kyoto Prefectural University of Medicine, Kyoto, 602-8566, Japan; ^4^ Japan Institute for Food Education and Health, St. Agnes’ University, Kyoto, 602-8013, Japan

**Keywords:** AE1, esophageal squamous cell carcinoma, MAPK, Hedgehog signaling pathway, cellular physiology

## Abstract

Recent studies have described important roles for the anion exchanger (AE) in epithelial carcinogenesis and tumor behavior. The objectives of the present study were to investigate the role of AE1 in the regulation of genes involved in tumor progression and the clinicopathological significance of its expression in esophageal squamous cell carcinoma (ESCC). An immunohistochemical analysis was performed on 61 primary tumor samples obtained from ESCC patients who underwent esophagectomy. AE1 was primarily located in the cell membranes or cytoplasm of carcinoma cells, and its distribution pattern was related to the histological degree of the differentiation of SCC or the pT category. Among patients with pT2-3 ESCC, the 5-year survival rate of patients with diffuse AE1 expression (40.2%) was significantly lower than that of patients with focal expression (74.0%). AE1 was strongly expressed in KYSE150 and TE8 human ESCC cells. The depletion of AE1 using siRNA inhibited cell proliferation, migration, and invasion and induced apoptosis. The results of the microarray analysis revealed that MAPK and Hedgehog signaling pathway-related genes, such as DHH, and GLI1, were down-regulated in AE1-depleted KYSE150 cells. In conclusions, the results of the present study suggest that the diffuse expression of AE1 is related to a worse prognosis in patients with advanced ESCC, and that it regulates tumor progression by affecting MAPK and Hedgehog signaling pathways. These results provide an insight into the role of AE1 as a mediator of and/or a biomarker for ESCC.

## INTRODUCTION

The anion exchanger (AE) is a transmembrane protein that exchanges chloride (Cl^-^) for bicarbonate (HCO_3_^-^) via the cell membrane, and is involved in the regulation of transepithelial ion transport and maintenance of intracellular pH [1, 2, 3]. Three isoforms of the AE have been identified to date: AE1, AE2, and AE3. Although these isoforms differ in their cytoplasmic (N-terminal) and membrane-spanning domains (C-terminal), their roles in anion transport are similar [1, 4, 5]. The expression of AE2 is reportedly ubiquitous, whereas that of AE1 is restricted to the erythrocyte cell membrane and basolateral surface of alpha-intercalated cells in the collecting duct of the kidney. AE3 is expressed in the brain, retina, and heart [3, 6].

Several recent studies described important roles for AE in tumorigenesis, differentiation, survival, and invasion [7–11]. AE1 was unexpectedly found to be expressed and important for cell cycle progression in gastric and colonic cancers, and high AE1 levels have been associated with a poor prognosis [3, 5]. The small interfering RNA (siRNA)-mediated suppression of AE1 has inhibited the growth of gastric carcinoma in *in vitro* [3, 5, 8, 9] and *in vivo* studies [12], indicating its potential as a target for cancer therapy. However, the roles of AE1 in the carcinogenesis, development, and progression of esophageal squamous cell carcinoma (ESCC) remain unclear. Moreover, the clinical significance of AE1 expressing in human ESCC has not yet been examined.

The aims of this research were to determine the roles of AE1 in the control of tumorigenesis related genes and its clinical meaning in esophageal cancer. By analyzing the AE1 expression in human ESCC tissues, relationships with the clinicopathological features and prognosis of ESCC patients were investigated. In addition, microarray data revealed that the knocking down with AE1 siRNA affected a lot of genes related to mitogen-activated protein kinase (MAPK) and Hedgehog signaling.

## RESULTS

### Immunohistochemical analysis of AE1 expression in ESCC tumors

**Figure 1:**
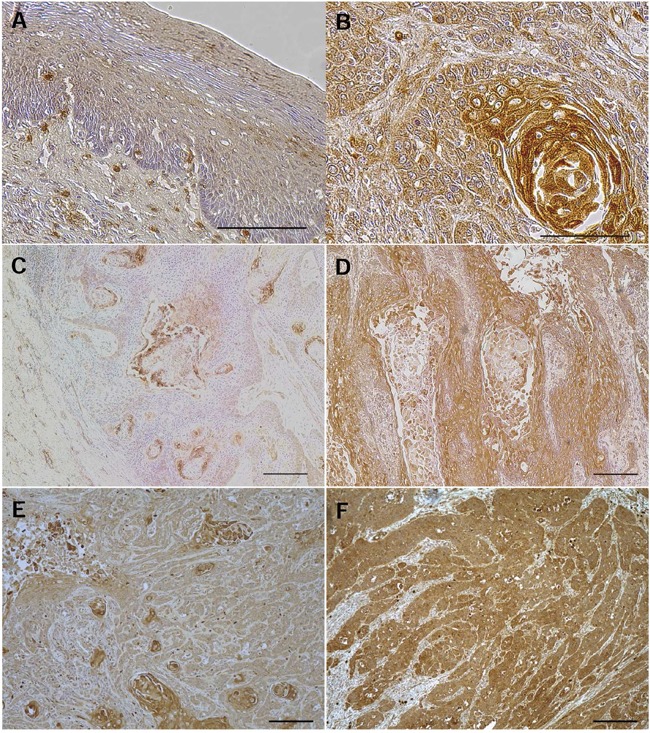
AE1 protein expression in human ESCCs **A**. Immunohistochemical staining of human esophageal epithelia with an AE1 antibody. Cells expressing AE1 were primarily confined to the lower and middle layers of the squamous epithelium, with the exception of the basal and parabasal cell layers. Magnification: ×400. Bar 100 μm. **B**. Immunohistochemical staining of primary human ESCC samples with the AE1 antibody. Magnification: ×400. Bar 100 μm. **C**. Immunohistochemical staining of primary human ESCC samples with the low grade expression of AE1. Magnification: ×100. Bar 200 μm. **D**. Immunohistochemical staining of primary human ESCC samples with the high grade expression of AE1. Magnification: ×100. Bar 200 μm. **E**. Immunohistochemical staining of primary human ESCC samples with focal AE1 expression. Magnification: ×100. Bar 200 μm. **F**. Immunohistochemical staining of primary human ESCC samples with diffuse AE1 expression. Magnification: ×100. Bar 200 μm.

An immunohistochemistry for the AE1 protein revealed that AE1 expression was mainly observed in the lower and middle layer of the non-cancerous esophageal epithelia, and not detected in the basal and para-basal cell layers (Figure 1A). In ESCC tissues, the AE1 protein was chiefly expressed in the cell membranes or cytoplasm of cancer cells (Figure 1B). The median AE1 score was 1.8 (range=0-2.2; mean±standard deviation (SD) = 1.54±0.60), and patients were categorized into low (scores <1.8, n=28) and high expression groups (scores≥1.8, n=33) (Figure 1C–1D, Supplementary Figure 1A–1B). In the analysis of their clinicopathological features, the AE1 expression correlated with tumor length (Table 1).

**Table 1:** Relationships between clinicopathological features of ESCC and expression of AE1

Variable	Staining score		p value	Distribution		p value
	Low (n=28)	High (n=33)		Focal (n=22)	Diffuse (n=39)	
Gender						
Male	24	28	0.9243	19	33	0.8533
Female	4	5		3	6	
Age						
<65 years	16	21	0.6049	13	24	0.851
≥65 years	12	12		9	15	
Tumor length						
<50 mm	22	18	0.0491*	14	26	0.811
≥50 mm	6	15		8	13	
Histological type						
Well/moderately differentiated SCC	19	25	0.4928	20	24	0.0140*
Poorly differentiated SCC	9	8		2	15	
Lymphatic invasion						
Negative	12	16	0.6603	9	19	0.5567
Positive	16	17		13	20	
Venous invasion						
Negative	15	21	0.4257	13	23	0.9929
Positive	13	12		9	16	
pT						
pT1	15	12	0.1775	6	21	0.0448*
pT2-3	13	21		16	18	
pN						
pN0	11	16	0.471	13	14	0.0799
pN1-3	17	17		9	25	

SCC: squamous cell carcinoma; pT: pathological T stage; pN: pathological N stage.

*p<0.05: chi-squared test.

Next, we focused on the pattern of the distribution of AE1-expressing cells. Patients were categorized into 2 groups based on the distribution pattern: focal AE1 expression (n=22) and diffuse AE1 expression (n=39) (Figure 1E–1F, Supplementary Figure 1C–1D). In the analysis of their clinicopathological features, relationships were observed between the distribution pattern of AE1 and the histological degree of the differentiation of SCC or pT category (Table 1). Regarding pN category, frequency of lymph node metastasis tended to be higher in patents with diffuse AE1 expression (64.1%) than those with focal expression (40.9%) without significant difference (Table 1).

**Figure 2:**
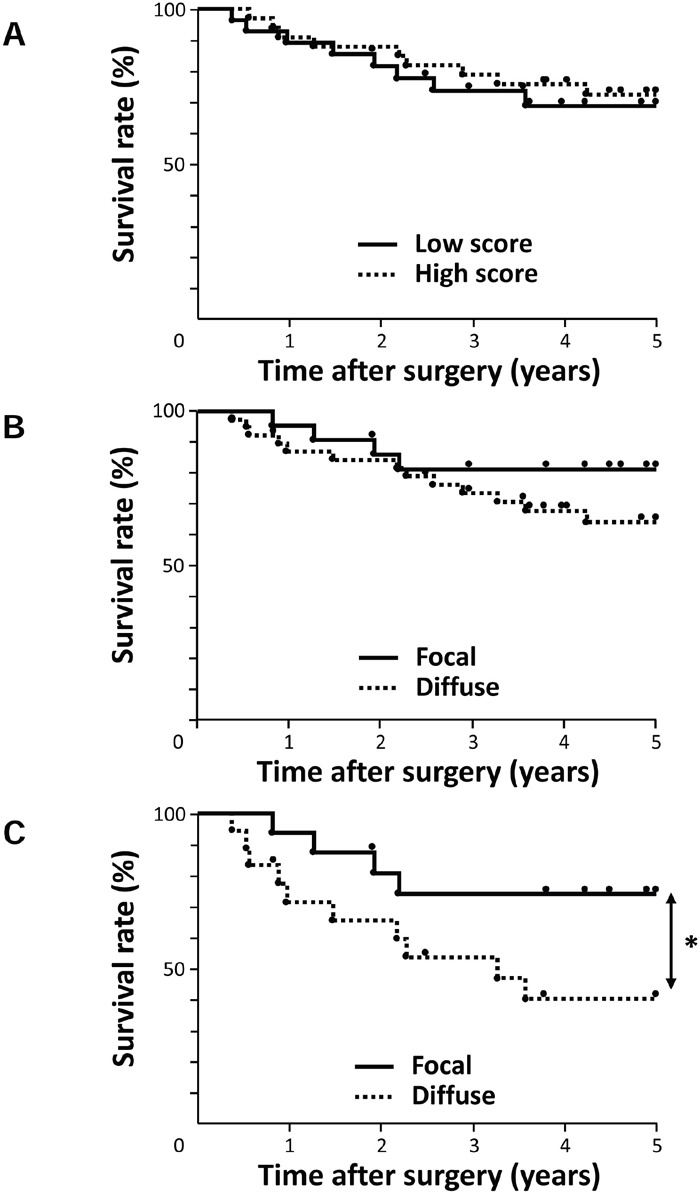
Survival curve of patients after curative resection for ESCC according to the expression of AE1 **A**. All patients were classified into two groups: the low grade expression of AE1 (n=28) and high grade expression of AE1 (n=33) in the tumor. **B**. All patients were classified into two groups: focal AE1 expression (n=22) and diffuse AE1 expression (n=39) in the tumor. **C**. Patients with pT2-3 ESCC were classified into two groups: focal AE1 expression (n=16) and diffuse AE1 expression (n=18). *p<0.05: Log-rank test.

We then investigated the prognostic significance of AE1 expression after curative resection. We determined which of the 10 variables (gender, age, tumor length, histological degree of the differentiation of SCC, lymphatic invasion, venous invasion, pT and pN categories, AE1 staining, and AE1 distribution) influenced survival. A univariate analysis showed that venous invasion, the pT and pN categories significantly correlated with prognosis (p=0.033, 0.002, and 0.032, respectively) (Table 2). The AE1 staining score itself did not correlate with prognosis (Figure 2A, Table 2). Regarding the distribution pattern of AE1, the 5-year survival rate of the diffuse AE1 expression group (64.3%) was poorer than that of the focal AE1 expression group (81.3%), but not significantly (p = 0.176) (Figure 2B, Table 2).

**Table 2:** Five-year survival rate of patients with ESCC according to various clinicopathological parameters

Variable	All patients (n=61)		pT2-3 (n=34)	
	5-year survival rate (%)	p value	5-year survival rate (%)	p value
Gender				
Male	69.43	0.968	51.14	0.4023
Female	76.19		83.33	
Age				
<65 years	68.44	0.6281	59.58	0.9227
≥65 years	73.23		51.95	
Tumor length				
<50 mm	70.64	0.7859	50.42	0.6342
≥50 mm	70.83		63.73	
Histological type				
Well/moderately differentiated SCC	73.13	0.2334	58.01	0.4077
Poorly differentiated SCC	63.73		53.03	
Lymphatic invasion				
Negative	80.54	0.1361	61.9	0.4079
Positive	61.66		52.63	
Venous invasion				
Negative	81.59	0.0327*	72.32	0.2341
Positive	55.38		44.44	
pT				
pT1	87.72	0.0024*		
pT2-3	56.72			
pN				
pN0	84.74	0.0318*	64.17	0.3589
pN1-3	59.65		51.98	
AE1 staining score				
Low	68.67	0.8276	36.26	0.1469
High	72.31		66.67	
AE1 distribution				
Focal	81.34	0.1761	74.04	0.0387*
Diffuse	64.31		40.18	

SCC: squamous cell carcinoma; pT: pathological T stage; pN: pathological N stage.

*p<0.05: Log-rank test.

Further, we investigated the prognostic impact of the AE1 expression in accordance with the pT category because it correlated with the distribution pattern of AE1 in Table 1. In patients with pT1 ESCC, a univariate analysis revealed that only the pN category was a significant prognostic factor (p=0.046) (Supplementary Table 1). Regarding the distribution pattern of AE1 in pT1 cases, the 5-year survival rate of the diffuse AE1 expression group (84.0%) was lower than that of the focal AE1 expression group (100%), but not significantly (p = 0.332) (Supplementary Table 1, Supplementary Figure 2). In patients with pT2-3 ESCC, the distribution pattern of AE1 was the strongest prognostic factor (p=0.039) (Table 2). Among pT2-3 cases, the 5-year survival rate of the diffuse AE1 expression group (40.2%) was significantly poorer than that of the focal AE1 expression group (74.0%) (Figure 2C). Multivariate analysis with factors whose p-values were less than 0.500 in univariate analysis also revealed the distribution pattern of AE1 was the most important prognostic indicator (Supplementary Table 2), suggesting that the diffuse AE1 expression is a valid poor prognostic indicator for advanced esophageal cancer.

In regard to recurrent pattern after curative resection, the number of cases with hematogenous recurrence was significantly larger in the diffuse AE1 expression group than in the focal AE1 expression group, although there was no difference in the percentage of postoperative adjuvant therapy [13] (Supplementary Table 3). We then analyzed 31 patients performed postoperative adjuvant therapy. In the diffuse AE1 expression group, 1 patient (5.3%) had hematogenous recurrence, whereas there were no patients with hematogenous recurrence in the focal AE1 expression group. Further, the number of patients with lymphogenous recurrence was larger in the diffuse AE1 expression group (n=8, 42.1%) than in the focal AE1 expression group (n=2, 16.7%) without significant difference (p=0.14).

### Expression of AE1 in ESCC cell lines

**Figure 3:**
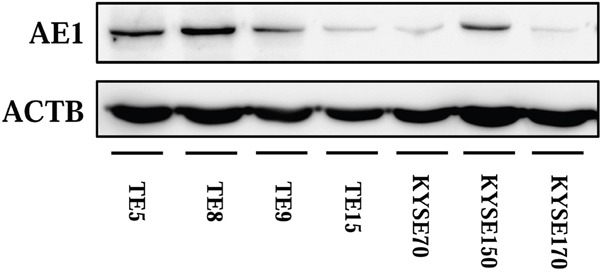
Expression of AE1 in ESCC cells AE1 protein expression was analyzed in 7 ESCC cell lines. Western blotting showed that AE1 was strongly expressed in TE5, TE8, TE9, and KYSE150 cells.

In order to elucidate functions of AE1 in ESCCs, we investigated 7 cell lines for AE1 protein expressions. Results of western blot revealed that AE1 was strongly expressed in TE5, TE8, TE9, and KYSE150 cell lines (Figure 3).

### AE1 regulates cell cycle in ESCC cells

**Figure 4:**
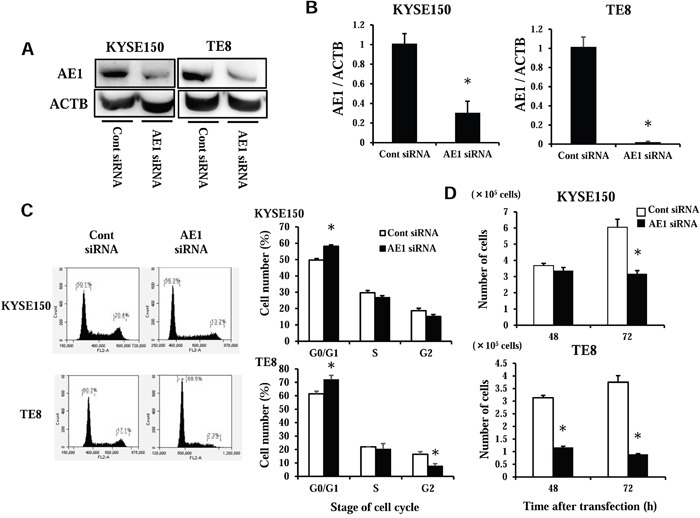
AE1 controls the cell cycle progression of ESCC cells **A**. Western blotting revealed that AE1 siRNA effectively reduced AE1 protein levels in KYSE150 and TE8 cells. **B**. AE1 siRNA effectively reduced AE1 mRNA levels in KYSE150 and TE8 cells. Mean ± SEM. n = 3. *p<0.05 (significantly different from control siRNA). **C**. The down-regulation of AE1 partially reduced cell cycle progression from the G_1_ to S phase in KYSE150 and TE8 cells. Cells transfected with control or AE1 siRNA were stained with propidium iodide (PI) and analyzed by flow cytometry. Mean ± SEM. n = 3. *p<0.05 (significantly different from control siRNA). **D**. The down-regulation of AE1 inhibited the proliferation of KYSE150 and TE8 cells. The number of cells was counted 48 and 72 h after siRNA transfection. Mean ± SEM. n = 4. *p<0.05 (significantly different from control siRNA).

We performed knocking down experimentation with AE1 siRNA in KYSE150 and TE8 cell lines and investigated the influences on cell cycle regulation. AE1 protein and mRNA levels were obviously decreased by AE1 siRNA transfection in both cell lines (Figure 4A–4B). The knocking down of AE1 partially inhibited cell cycle process from the G_1_ to S phase in both KYSE150 and TE8 cells (Figure 4C). Cell number 72 h after transfection was lower in AE1 siRNA transfected KYSE150 cells than in control cells with significant differences (Figure 4D). In TE8 cells, the cell number of AE1 knockdown cells was significantly lower than those of control cells 48 h and 72 h after transfection (Figure 4D). We also conducted overexpression study. Cells transfected Control-HaloTag® plasmid and AE1-HaloTag® plasmid were stained in red (Supplementary Figure 3A), and AE1-HaloTag® plasmid increased AE1 mRNA levels (Supplementary Figure 3B). AE1 overexpression in KYSE150 cells and TE8 cells increased cell growth (Supplementary Figure 4A) as opposed to knockdown of AE1. These findings indicate that AE1 has a critical function in control of cell cycle and proliferation in esophageal cancer.

### AE1 regulates apoptosis in ESCC cells

**Figure 5:**
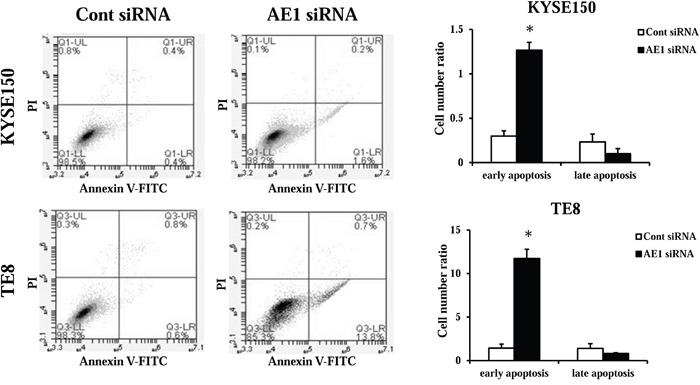
AE1 controls the survival of ESCC cells The down-regulation of AE1 induced cell death in KYSE150 and TE8 cells. Apoptosis was determined by flow cytometry using PI/annexin V double staining. Mean ± SEM. n = 3. *p<0.05 (significantly different from control siRNA).

Next, we transfected KYSE150 and TE8 cells with AE1 siRNA and examined apoptosis. The AE1depletion increased early apoptosis (annexin V positive/PI negative) in KYSE150 and TE8 cell lines 48 h after siRNA transfection (Figure 5). These findings suggest that the expression of AE1 affects cell survival in ESCC cells.

### AE1 controls cell migration and invasion in ESCC cells

**Figure 6:**
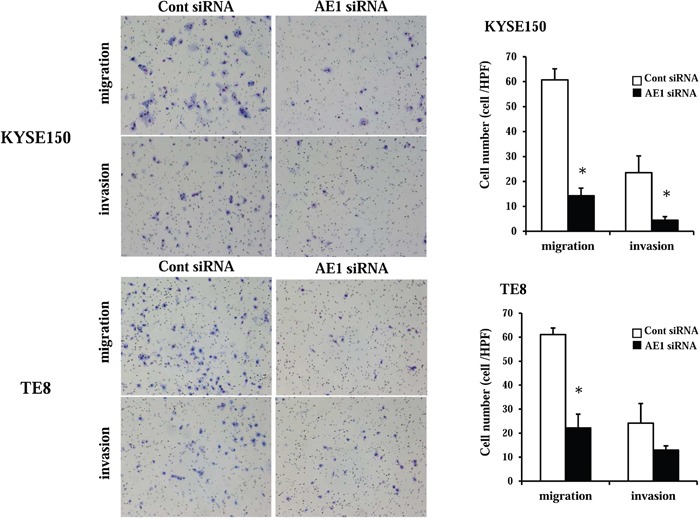
AE1 controlled the migration and invasion of ESCC cells The down-regulation of AE1 inhibited the migration and invasion of KYSE150 and TE8 cells. Cell migration and invasion were examined using the Boyden chamber assay. Mean ± SEM. n = 3. *p<0.05 (significantly different from control siRNA).

In KYSE150 cells, AE1 siRNA significantly reduced cell migration and invasion (Figure 6). In TE8 cells, the AE1 depletion also reduced cell migration and invasion (Figure 6). AE1 overexpression in TE8 cells increased cell invasion (Supplementary Figure 4B) as opposed to knockdown of AE1. These findings indicate that AE1 has critical functions in the control of cell migration and invasion in esophageal cancer.

### Microarray analysis in AE1 siRNA-transfected ESCC cells

We determined the gene expression profiling of AE1 siRNA-transfected KYSE150 cells in microarray analysis. The results revealed that the expressions of 3345 genes showed fold changes of > 2.0 in KYSE150 cell line upon the knockdown of AE1. Among these, 588 were up-regulated and 2757 were down-regulated in AE1 siRNA-transfected KYSE150 cell line. Supplementary Table 4 showed the list of 20 genes with expression levels strongly up- or down-regulated in AE1 siRNA-transfected KYSE150 cell line. Ingenuity Pathway Analysis (IPA) revealed “Cancer” was one of the top-ranking diseases and that “Cellular Movement”, “Cell Death and Survival”, “Cell Cycle”, “Cellular Growth and Proliferation” were top-ranking biological functions related to AE1 depletion (Supplementary Table 5). A list of the top 50 up- or down-regulated genes exhibiting cell proliferation, cell cycle, apoptosis, migration, and invasion-related functions is shown in Supplementary Table 6, and was consistent with the results obtained in our *in vitro* studies.

### Signal pathways and molecular mechanisms regulated by AE1 in ESCC cells

Pathway analysis using IPA showed that “P38 MAPK” was the center in one of the top-ranking signal networks of AE1 functions (Supplementary Figure 5). Furthermore, in Supplementary Table 5, “Molecular Mechanisms and Cancer” was the top-ranking canonical pathway related to the depletion of AE1. An analysis of the map of this pathway revealed that Hedgehog pathway-related genes were strongly down-regulated by the depletion of AE1 (Supplementary Figure 6A–6B), and also that MAPKs, such as “P38 MAPK”, “JNK”, and “ERK”, were included in this map. Therefore, we focused on MAPK and Hedgehog signaling pathways, and analyzed functions of AE1 in the control of these pathways.

**Figure 7:**
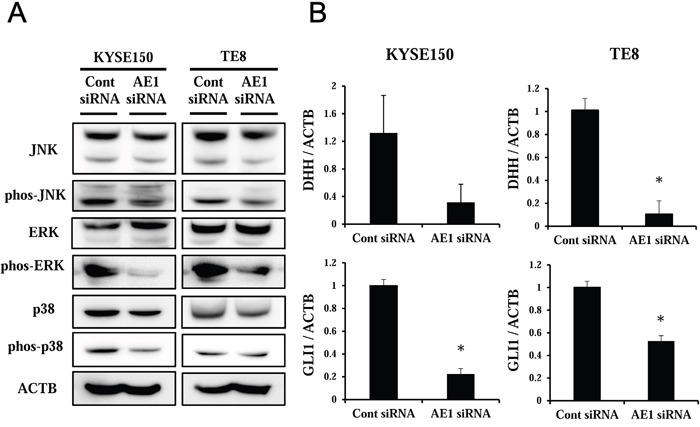
Signal pathways regulated by AE1 in ESCC cells **A**. The down-regulation of AE1 decreased the phosphorylation levels and/or the total protein levels of MAPKs, such as JNK, ERK, and p38, in KYSE150 and TE8 cells. **B**. Verification of gene expression by real-time quantitative RT-PCR. The expression levels of two selected Hedgehog signaling pathway-related genes (DHH and GLI1) in AE1-depleted KYSE170 and TE8 cells were compared to those in control siRNA-transfected cells using real-time quantitative RT-PCR. Mean ± SEM. n = 3. *p<0.05 (significantly different from control siRNA).

The gene expression profiles of AE1-depleted KYSE150 cells showed that several genes of MAPKs were down-regulated by the knockdown of AE1 (Table 3). A Western blot analysis revealed that the down-regulation of AE1 decreased the phosphorylation levels and/or the total protein levels of JNK, ERK, and p38 in KYSE150 and TE8 cells (Figure 7A, Supplementary Figure 7).

**Table 3:** MAPK and Hedgehog signaling pathway-related genes with expression levels in KYSE150 cells that were changed by the depletion of AE1

**MAPK signaling pathway**			
**Symbol**	**Gene Name**	**UniGene ID**	**Exp Fold Change**
MAPK11	mitogen-activated protein kinase 11 (p38-β)	Hs.57732	−4.599
MAPK12	mitogen-activated protein kinase 12 (p38-γ)	Hs.432642	−3.629
MAPK6	mitogen-activated protein kinase 6 (ERK3)	Hs.411847	−2.668
MAPK3	mitogen-activated protein kinase 3 (ERK1)	Hs.861	−2.475
MAPK9	mitogen-activated protein kinase 9 (JNK2)	Hs.484371	−2.403
MAP3K5	mitogen-activated protein kinase kinase kinase 5	Hs.186486	−2.235
**Hedgehog signaling pathway**			
**Symbol**	**Gene Name**	**UniGene ID**	**Exp Fold Change**
DHH	desert hedgehog	Hs.524382	−247.87
GLI1	GLI family zinc finger 1	Hs.632702	−8.354
PTCH1	patched 1	Hs.494538	−2.597
STK36	serine/threonine kinase 36	Hs.471404	−2.234
SUFU	suppressor of fused homolog	Hs.404089	−2.177

The results of the microarray analysis showed that several Hedgehog signaling pathway-related genes were down-regulated in AE1-depleted KYSE150 cells (Table 3). In order to verify the data of gene expression profiling, the 2 top-ranking genes (DHH and GLI1) were examined further by quantitative RT-PCR. The expression levels of DHH and GLI1 mRNA were decreased by the AE1 siRNA-transfection in KYSE150 cells (Figure 7B). Similar results were obtained in the TE8 cell line (Figure 7B).

These results were consistent with those of the gene expression profiling and indicate MAPK and Hedgehog signaling pathways are key mechanisms by which AE1 controls cancer cell functions, such as the proliferation, survival, and cellular movement of ESCC cells.

## DISCUSSION

Regarding AE1 expression in human carcinoma samples using an immunohistochemical examination, a previous report showed it correlated with tumor length, depth, lymph node metastasis, and prognosis in gastric carcinoma [3]. Here, we analyzed AE1 expression in ESCC and examined relationships with clinical backgrounds and prognoses. We found a correlation between the expression of AE1 and tumor length. Furthermore, a correlation was observed between the distribution pattern of AE1 and histological degree of the differentiation of SCC or pT category. Our results showed the diffuse AE1 expression might become an effective poor prognostic indicator for advanced esophageal cancer. On the other hand, we need to state the limitation of this retrospective study related to small sample size and selection bias because our eligibility criteria was no preoperative chemotherapy. In fact, preoperative therapy has been performed positively for advanced ESCC in Japan [14]. However, as far as we know, this is the first report to investigate the expression of AE1 in human ESCC samples and its gene expression profiling.

Regarding mechanisms by which AE1 plays a role in carcinogenesis, previous studies demonstrated that AE1 binds with p16 and influences cell cycle progression [3, 5, 15]. A recent study showed that the transfection with miR-24 induced the return of p16 to the nucleus, confirming the miR-24-controlled AE1 down-regulation in gastric carcinoma [8]. Furthermore, AE1 expression in gastric carcinoma is associated with cellular alkalization, which plays a role in carcinogenesis [3, 5, 8, 12]. Here, we identified that the distribution pattern of AE1 correlated with the pT factor, which suggested AE1 was diffusely expressed from the initial stage of oncogenesis. The distribution pattern of AE1 also correlated with degree of the differentiation of SCC, and in non-cancerous esophageal epithelia, cells expressing AE1 were mainly observed in the lower and middle layer, and not detected in the basal and para-basal layers. These suggest that cancer cells may require differentiation and/or hypoxia-inducible AE1 distribution. In addition, the present study revealed the diffuse AE1 expression was the most critical poor prognostic indicator in pT2-3 esophageal cancer. Several reports including our previous study demonstrated that the gene expression of hypoxia-inducible factors was elevated with the overexpression of pH regulators, such as carbonic anhydrase (CA), suggesting that they become effective poor prognostic factors in microenvironment where the expression of hypoxia-inducible factors is also increased [16, 17]. However these mechanisms should be determined in more detail in further investigations, the present study indicates the critical and specific functions of AE1 in advanced esophageal cancer. In addition, based on the results of gene expression profiles in this report, we newly discovered that MAPK and Hedgehog signaling pathways are important networks regulated by AE1.

The Hedgehog signaling is the important controlling element during embryonic development, and is involved in cellular functions, such as patterning, proliferation, and differentiation [18]. Mammals have three Hedgehog homologues: Sonic, Indian, and Desert Hedgehog (DHH). Canonical Hedgehog signal activation is caused by the interaction between Hedgehog ligands and the transmembrane protein receptor patched (PTCH). In the presence of Hedgehog ligands, PTCH reduces the suppression on the smoothened (SMO), leading to the activation of GLI transcription factors [18]. GLI increases the expression of various target genes, including controlling elements of fundamental cellular functions, and, thus, the Hedgehog signaling pathway functions in tumor progression. In esophageal carcinoma, previous reports showed that Hedgehog signaling is active and required for tumor growth [19, 20]. The results of the present study indicated the gene expression of these important factors in the Hedgehog signaling pathway, such as DHH, GLI1, and PTCH1, was changed by the knockdown of AE1, suggesting that AE1 regulates the tumor behavior of ESCC via this pathway.

Our results also revealed that the depletion of AE1 inhibited the activity of MAPKs in ESCC. In Barrett's esophageal adenocarcinoma cells, AE was shown to regulate MAPK-mediated proliferation via intracellular acidification [7]. Furthermore, recent studies identified crosstalk between Hedgehog and MAPK signaling in various types of tumors [18]. In human ESCC samples, the phosphorylation of ERK was detected in samples strongly expressing SHH and GLI1 [21]. In ESCC, Hedgehog signaling-induced ERK activation was previously shown to be repressed by PD98059 and cyclopamine [21]. An activation of Sonic Hedgehog enhanced proliferation, and this phenomenon was inhibited by a pre-incubation with cyclopamine and also by PD98059, suggesting that inactivation of ERK reduced the Hedgehog signaling-induced proliferation of ESCC cells [21]. These findings show that crosstalk between MAPK and Hedgehog signaling pathways exists in ESCC, and our results suggest novel and crucial roles for AE1 in this important cross-signaling.

Latest researches indicated ion transporters have critical functions in various cancer cells, and a cellular physiological factors are expected to become a novel and effective targets for tumor therapies [22, 23]. Our previous researches demonstrated the importance of ion carriers [24, 25], water transporters [26], and pH controlling factors [16] in ESCC. Several reports show the intracellular chloride concentration ([Cl^-^]_i_) controlled by Cl^-^ transporters may become the important messenger [27–29]. Our previous reports revealed that the change of the [Cl^-^]_i_ induced cell cycle arrest at the G_0_/G_1_ phase, and that the [Cl^-^]_i_ controls the proliferation by affecting MAPKs in cancer cells [27, 28]. AE1 is the key molecule regulating the [Cl^-^]_i_ through the exchange of Cl^-^ with HCO_3_^-^ across the plasma membrane, suggesting the mechanism by which AE1 plays a role in activation of MAPKs. In addition, regarding pH regulators, we showed that CA XII, which is involved in the acidification of circumstances, was an effective prognostic indicator for advanced esophageal cancer [16]. Suo et al. demonstrated that the *in vivo* delivery of siRNA resulted in the selective inhibition of AE1 expression, leading to a decreased incidence of gastric cancer in mice [12]. These findings suggest that pH regulatory factors, such as AEs, and CAs, lead the possibility to become effective treatment targets and their regulation may produce novel strategies for futurity therapies [22, 23].

In summary, we herein demonstrated that AE1 played as a regulator of the proliferation, survival, migration, and invasion of ESCC cell lines. The results of an immunohistochemistry indicated that the diffuse AE1 expression was a valid poor prognostic indicator for advanced esophageal cancer. Our microarray analysis also suggests that AE1 markedly influences the gene expressions associated with the crosstalk between MAPK and Hedgehog signaling pathways. More profound investigations about functions of AE1 may increase its potential as one of the key biomarkers and targets of treatment for esophageal cancer.

## MATERIALS AND METHODS

### Cell lines, antibodies, and other reagents

The human ESCC cell lines TE5, TE8, TE9, and TE15 were obtained from the Riken Cell Bank (Tsukuba, Japan). The human ESCC cell lines KYSE70, KYSE150, and KYSE170 were obtained from the Japanese Collection of Research Bioresources Cell Bank (Osaka, Japan). These cells were grown in RPMI-1640 medium (Nacalai Tesque, Kyoto, Japan) supplemented with 100 U/ml of penicillin, 100 μg/ml of streptomycin, and 10% fetal bovine serum (FBS). Cells were cultured in flasks or dishes in a humidified incubator at 37°C under 5% CO_2_ in air.

The monoclonal anti-AE1 antibody used in the immunohistochemical analysis and protein assay was obtained from Abcam (Cambridge, MA, UK). The rabbit monoclonal c-Jun N-terminal kinase (JNK), phosphorylated JNK, extracellular signal-regulated kinase (ERK), phosphorylated ERK, p38, and phosphorylated p38 antibodies were purchased from Cell Signaling Technology (Beverly, MA). The mouse monoclonal ACTB antibody was purchased from Sigma-Aldrich (St. Louis, MO). Horseradish peroxidase (HRP)-conjugated anti-rabbit or mouse secondary antibodies were purchased from Cell Signaling Technology (Beverly, MA).

### Patients and primary tissue samples

ESCC tumor samples were obtained from 61 patients with histologically confirmed primary ESCC who underwent esophagectomy at Kyoto Prefectural University of Medicine between 1999 and 2009 and were embedded in paraffin after 12 h of formalin fixation. Patient eligibility criteria were as follows: no synchronous or metachronous cancers (in addition to ESCC) and no preoperative chemotherapy or radiation therapy. We excluded patients with non-curative resected tumors or non-consecutive data. All patients provided written informed consent. Relevant clinicopathological and survival data were obtained from the hospital database. Staging was principally based on the International Union Against Cancer (UICC)/TNM Classification of Malignant Tumors (7th edition) [30]. Cancer recurrence occurred in 24 patients (39.3%). Nineteen patients (31.1%) died of cancer recurrence, while no patients died from other diseases. The median follow-up period of all patients was 56.2 months (range, 4.5-157 months). With respect to the histological degree of the differentiation of SCC, patients were divided into 2 groups: well/moderately differentiated SCC and poorly differentiated SCC.

### Immunohistochemistry

Paraffin sections (thickness of 4 μm) of tumor tissues were subjected to immunohistochemical staining for the AE1 protein using the avidin-biotin-peroxidase method. Briefly, paraffin sections were dewaxed with xylene and hydrated with a graded series of alcohols. Endogenous peroxidases were quenched by incubating the sections for 30 min in 0.3% H_2_O_2_. For blocking of endogenous biotin, biotin receptors, and avidin binding sites, Avidin/Biotin Blocking Kit was used (Vector laboratories, Burlingame, CA). Sections were then treated with a protein blocker and incubated at 4°C overnight with the anti-AE1 antibody. The avidin-biotin-peroxidase complex (Vectastain ABC Elite kit; Vector Laboratories, Burlingame, CA) was visualized with diaminobenzidine tetrahydrochloride. Sections were counterstained with hematoxylin, dehydrated with a graded series of alcohols, cleared in xylene, and mounted.

Immunohistochemical samples stained with AE1 were graded semi-quantitatively by considering both the staining intensity and percentage of positive tumor cells using an immunoreactive score (IRS) [31]. Staining intensity was scored as 0 (no staining), 1 (weak staining), 2 (moderate staining), or 3 (strong staining). The proportion of positive tumor cells was scored from 0 to 1.0. The score of each sample was calculated as the maximum multiplied product of the intensity and proportion scores (0 to 3.0). Regarding the pattern of distribution of AE1-expressing cells, we divided ESCC patients into 2 groups: focal AE1 expression (lesion with mosaic pattern staining ≥50%) and diffuse AE1 expression (lesion with mosaic pattern staining<50%).

### Western blotting

Cells were harvested in M-PER lysis buffer (Pierce, Rockford, IL) supplemented with protease inhibitors (Pierce). Protein concentrations were measured with a modified Bradford assay (Bio-Rad, Hercules, CA). Cell lysates containing equal amounts of total protein were separated by SDS-PAGE and then transferred onto PVDF membranes (GE Healthcare, Piscataway, NJ). These membranes were then probed with the indicated antibodies, and proteins were detected using an ECL Plus Western Blotting Detection System (GE Healthcare). Band densities were quantified using the ImageJ software (http://rsb.info.nih.gov/ij/) after being scanned from the film.

### siRNA transfection

Cells were transfected with 12 nmol/l AE1 siRNA (Stealth RNAi™ siRNA #HSS185804; Invitrogen, Carlsbad, CA) using the Lipofectamine RNAiMAX reagent (Invitrogen), according to the manufacturer's instructions. Medium containing siRNA was replaced with fresh medium after 24 h. The control siRNA provided (Stealth RNAi™ siRNA Negative Control; Invitrogen) was used as a negative control.

### Overexpression study

Control-HaloTag® plasmid (Promega, G6591) and AE1-HaloTag® plasmid were transfected into KYSE150 cells and TE8 cells using FuGENE HD transfection reagents (Promega, E2311) following the manufacturer's instructions. Transfection of vector was confirmed by fluorescent microscopy for HaloTag® fusion protein stained with the TMR conjugated HaloTag® ligand (Promega, G8252) according to the manufacturer's protocol. Cells were then separated by flow cytometry using a Cell Sorter SH800 (SONY, Tokyo, Japan) based on fluorescence. After passaging cells, AE1-expressing cells were used for proliferation, migration and invasion assays.

### Cell cycle analysis

The cell cycle phase was evaluated 48 h after siRNA transfection by fluorescence-activated cell scoring (FACS). Briefly, cells were treated with Triton X-100 and RNase, and nuclei were stained with propidium iodide (PI) prior to the DNA content measurement using Becton-Dickinson Accuri C6 FACS (BD Biosciences, Franklin Lakes, NJ). At least 10,000 cells were analyzed.

### Cell proliferation

Cells were seeded on 6-well plates at a density of 1.0 × 10^5^ cells per well and incubated at 37°C with 5% CO_2_. siRNA was transfected 24 h after the cells had been seeded. Cells were detached from the flasks with trypsin-EDTA 48 h and 72 h after siRNA transfection and were counted using a hemocytometer.

### Analysis of apoptotic cells

Cells were harvested 48 h after siRNA transfection and stained with fluorescein isothiocyanate-conjugated annexin V and phosphatidylinositol using the annexin V kit (Beckman Coulter, Brea, CA) according to the manufacturer's protocol. The proportion of apoptotic cells was analyzed by flow cytometry with BD Accuri C6 (BD Biosciences).

### Analysis of cell migration and invasion

The migration assay was conducted using a Cell Culture Insert with a pore size of 8 μm (BD Biosciences). Biocoat Matrigel (BD Biosciences) was used to evaluate cell invasion potential. Briefly, cells (1.0 × 10^5^ cells per well) were seeded in the upper chamber in serum-free medium 24 h after siRNA transfection. The lower chamber contained medium with 10% FBS. The chambers were incubated at 37°C for 48 h in 5% CO_2_, and non-migrated or non-invaded cells were then removed from the upper side of the membrane by scrubbing with cotton swabs. Migrated or invaded cells were fixed on the membrane and stained with Diff-Quick staining reagents (Sysmex, Kobe, Japan). The migrated or invaded cells on the lower side of the membrane were counted in four independent fields of view at 100x magnification for each insert. Each assay was performed in triplicate.

### Real-time reverse transcription-polymerase chain reaction (RT-PCR)

Total RNA was extracted using an RNeasy kit (Qiagen, Valencia, CA). Messenger RNA (mRNA) expression was measured by quantitative real-time PCR (7300 Real-Time PCR System; Applied Biosystems, Foster City, CA) with TaqMan Gene Expression Assays (Applied Biosystems), according to the manufacturer's instructions. The expression levels of the following genes were measured: AE1 (Hs00978603_m1), DHH (Hs00368306_m1), and GLI1 (Hs00171790_m1) (Applied Biosystems). Expression was normalized for each gene to the housekeeping gene beta-actin (ACTB, Hs01060665_g1; Applied Biosystems). Assays were performed in triplicate.

### Microarray sample preparation and hybridization

Total RNA was extracted using an RNeasy kit (Qiagen). RNA quality was monitored with an Agilent 2100 Bioanalyzer (Agilent Technologies, Santa Clara, CA). Cyanine-3 (Cy3)-labeled cRNA was prepared from 0.1 μg of total RNA using a Low Input Quick Amp Labeling Kit (Agilent), according to the manufacturer's instructions. Samples were purified using RNeasy columns (Qiagen). A total of 0.60 μg of Cy3-labeled cRNA was fragmented and hybridized to an Agilent SurePrint G3 Human Gene Expression 8×60K Microarray for 17 h. Slides were washed and scanned immediately on an Agilent DNA Microarray Scanner (G2565CA) using the one color scan setting for 8×60K array slides.

### Processing of microarray data

Scanned images were analyzed with Feature Extraction Software 10.10 (Agilent) using default parameters to obtain background-subtracted and spatially detrended Processed Signal intensities. Signal transduction networks were analyzed using Ingenuity Pathway Analysis (IPA) software (Ingenuity Systems, Inc., Redwood City, CA).

### Statistical analysis

The chi-squared test was used to assess the differences between proportions, and Student's *t*-tests (for comparisons between two groups) were used to evaluate continuous variables. Survival curves were constructed by the Kaplan-Meier method, and differences in survival were examined using the Log-rank test. A multivariate analysis of the factors influencing survival was performed using the Cox proportional hazard model. Differences were considered significant when the relevant P value was <0.05. These analyses were performed using the statistical software JMP (version 10, SAS Institute Inc., Cary, NC).

## SUPPLEMENTARY MATERIALS FIGURES AND TABLES




