# Supplementary material for: Expression and role of anion exchanger 1 in esophageal squamous cell carcinoma
Source: Oncotarget. 2017 Jan 30;8(11):17921–35. doi: 10.18632/oncotarget.14900 (PMC5392297; doi:10.18632/oncotarget.14900)
Supplement: Supplementary file 1 [file oncotarget-08-17921-s001.pdf]

Expression and role of anion exchanger 1 in esophageal squamous cell carcinoma

SUPPLEMENTARY FIGURES AND TABLES

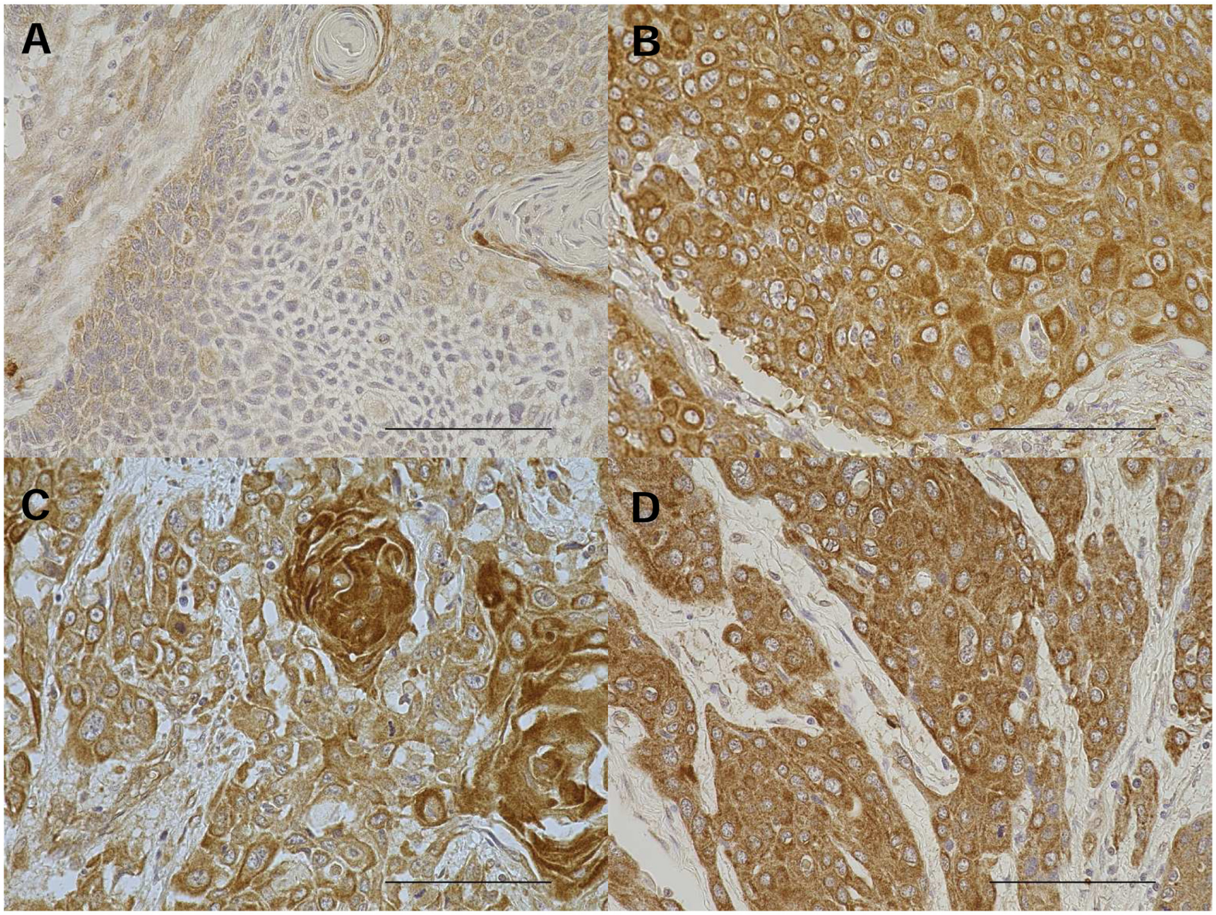

Supplementary Figure 1: AE1 protein expression in human ESCCs. A. Immunohistochemical staining of primary human ESCC samples with the low grade expression of AE1. Magnification: ×400. Bar 100 μm. **B.** Immunohistochemical staining of primary human ESCC samples with the high grade expression of AE1. Magnification: ×400. Bar 100 μm. **C.** Immunohistochemical staining of primary human ESCC samples with focal AE1 expression. Magnification: ×400. Bar 100 μm. **D.** Immunohistochemical staining of primary human ESCC samples with diffuse AE1 expression. Magnification: ×400. Bar 100 μm.

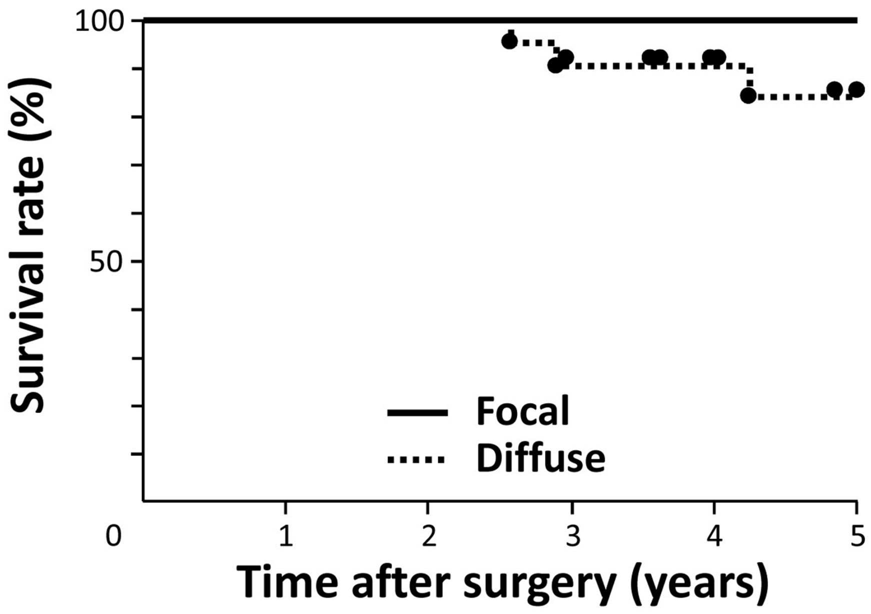

Supplementary Figure 2: Survival curve of patients with pT1 ESCC according to the expression of AE1. Patients with pT1 ESCC were classified into two groups: focal AE1 expression (n=6) and diffuse AE1 expression (n=21).

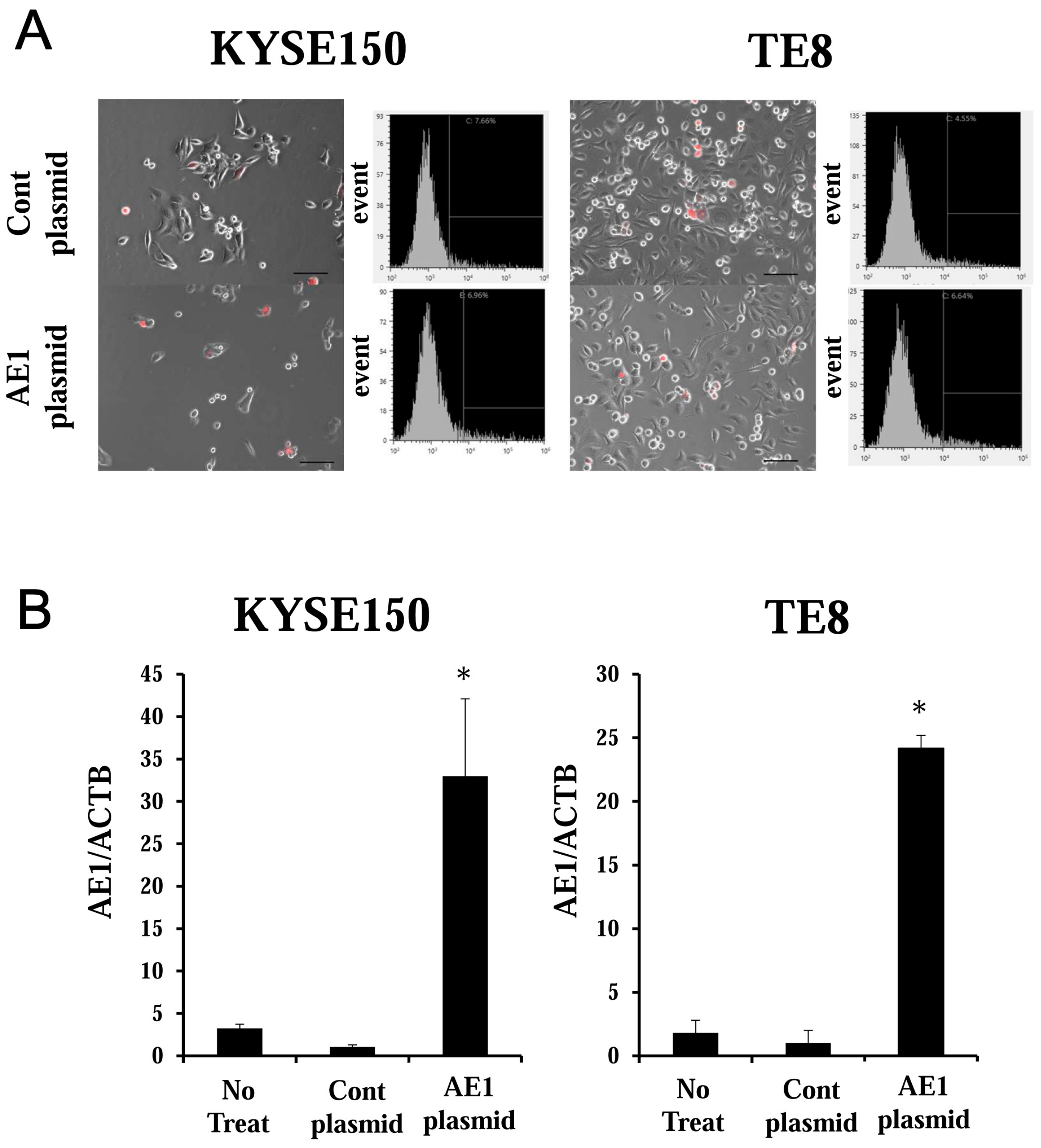

Supplementary Figure 3: AE1 overexpression in ESCC cells. A. Fluorescent microscopy for HaloTag® fusion protein. Cells transfected Control-HaloTag® plasmid and AE1-HaloTag® plasmid were stained in red. Magnification: ×10. Cells strongly expressing fluorescence were isolated via Fluorescence-Associated Cell Sorting. **B.** AE1 plasmid increased AE1 mRNA levels in KYSE150 and TE8 cells. Mean ± SEM. n = 3. *p<0.05 (significantly different from control plasmid).

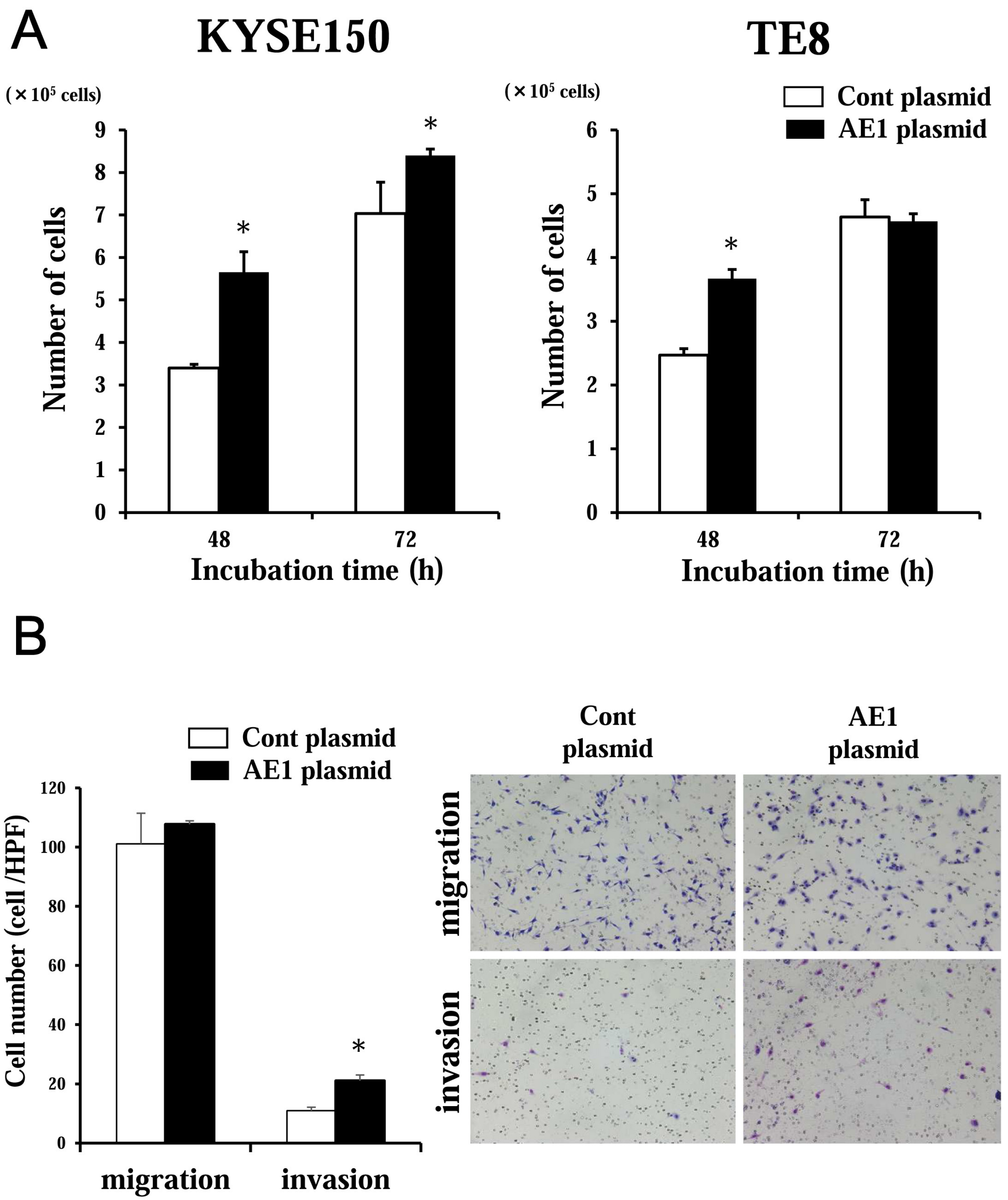

Supplementary Figure 4: AE1 overexpression increased cell proliferation and invasion in ESCC cells. A. The overexpression of AE1 increased cell proliferation in KYSE150 and TE8 cells. Mean ± SEM. n = 3. *p<0.05 (significantly different from control plasmid). **B.** The overexpression of AE1 increased cell invasion in TE8 cells. Mean ± SEM. n = 3. *p<0.05 (significantly different from control plasmid).

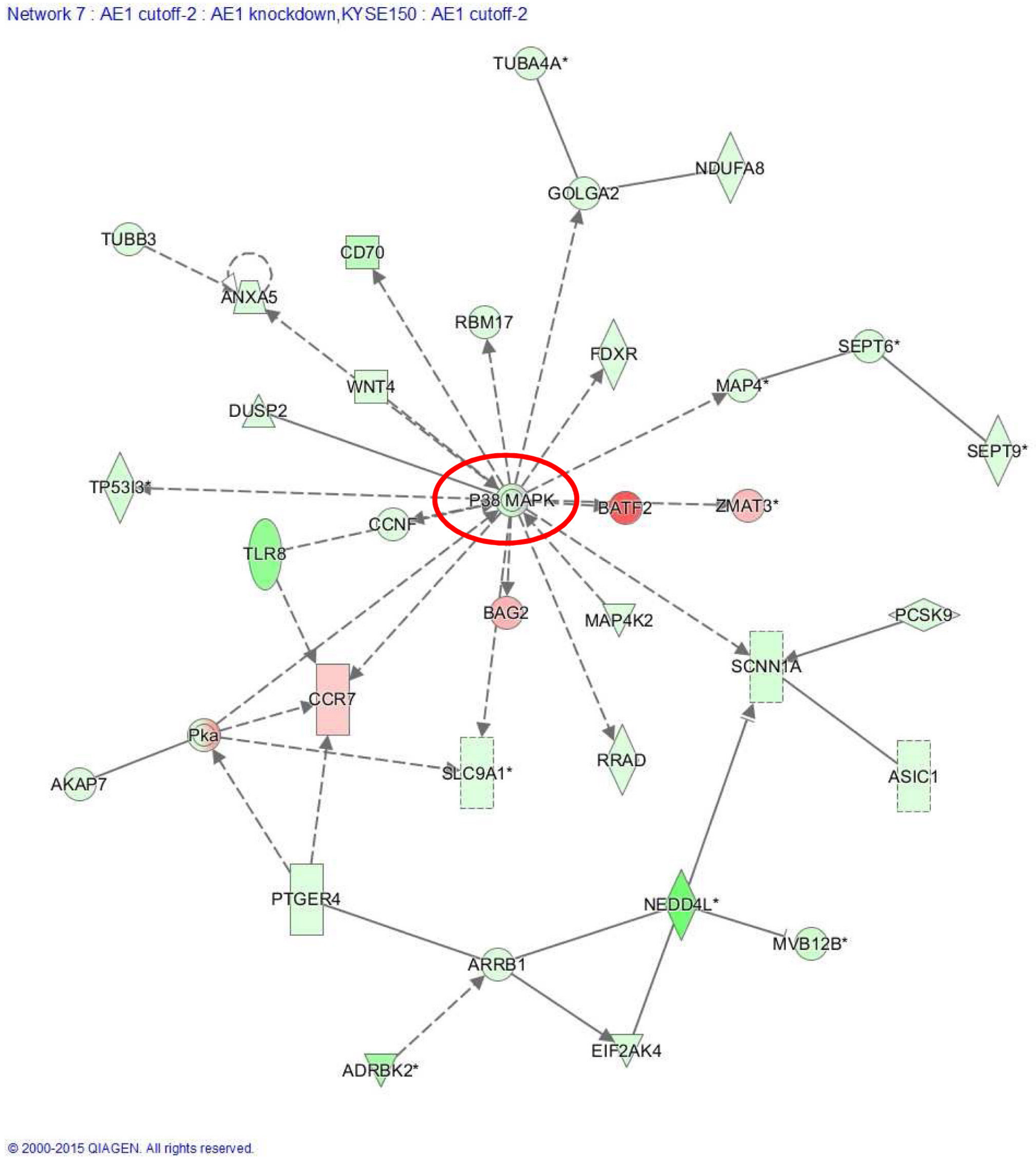

Supplementary Figure 5: The top-ranking signaling network related to the down-regulation of AE1 according to an Ingenuity Pathway Analysis. This network is called “Cancer, Organismal Injury and Abnormalities, Reproductive System Disease”. Red and green indicate genes with expression levels that were higher or lower, respectively, than reference RNA levels. MAPK-related genes were highlighted using red circles.

A

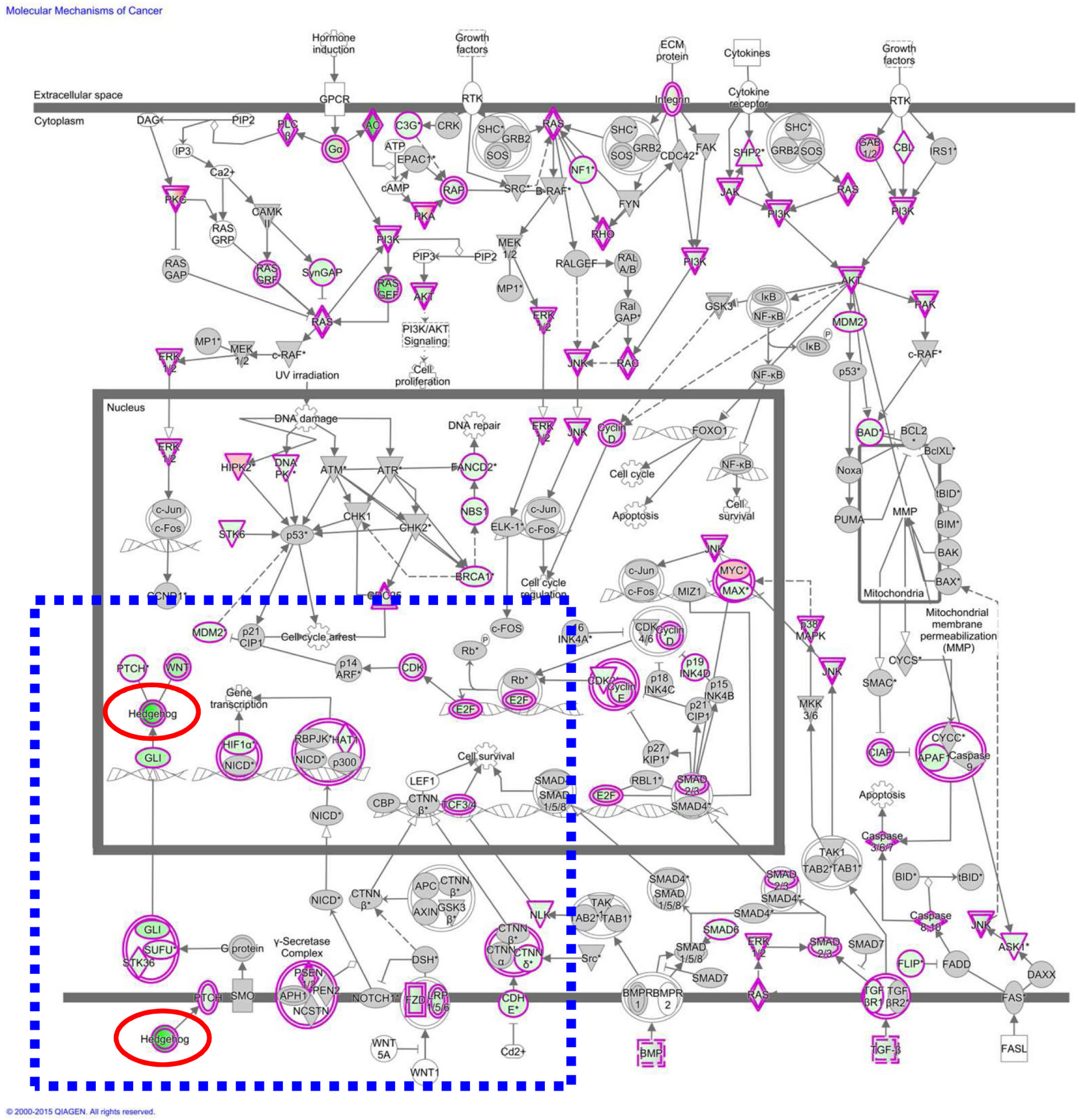

Supplementary Figure 6: The signaling map of “Molecular Mechanisms and Cancer”, the top-ranking canonical pathways related to AE1 depletion according to an Ingenuity Pathway Analysis. A. Red and green indicate genes with expression levels that were higher or lower, respectively, than reference RNA levels. Hedgehog signaling pathway-related genes were highlighted using red circles. MAPKs, such as “P38 MAPK”, “JNK”, and “ERK”, were also included in this map.

(*Continued*)

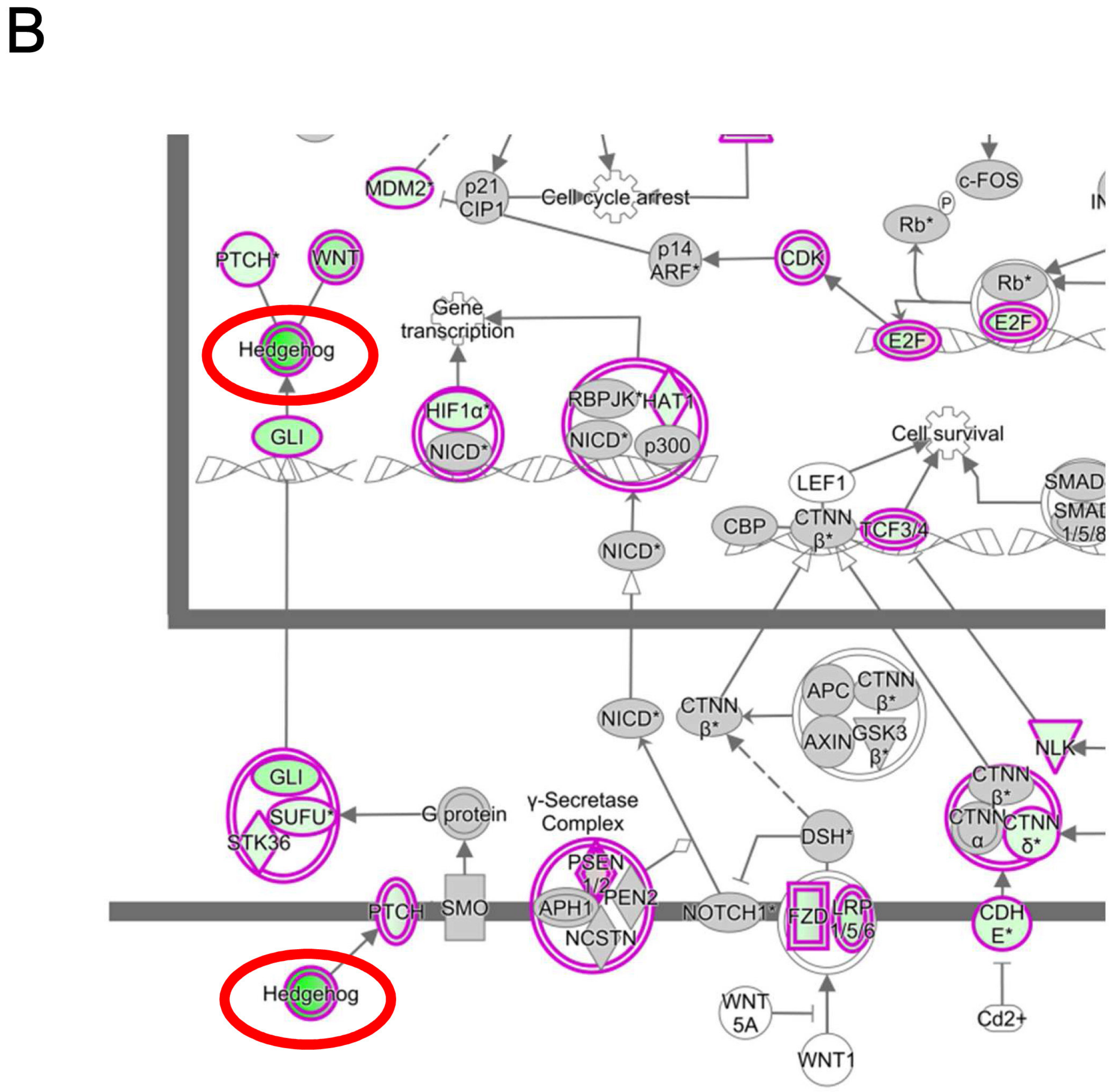

Supplementary Figure 6 (*Continued*): B. The highlighted part using blue square in (A) was magnified.

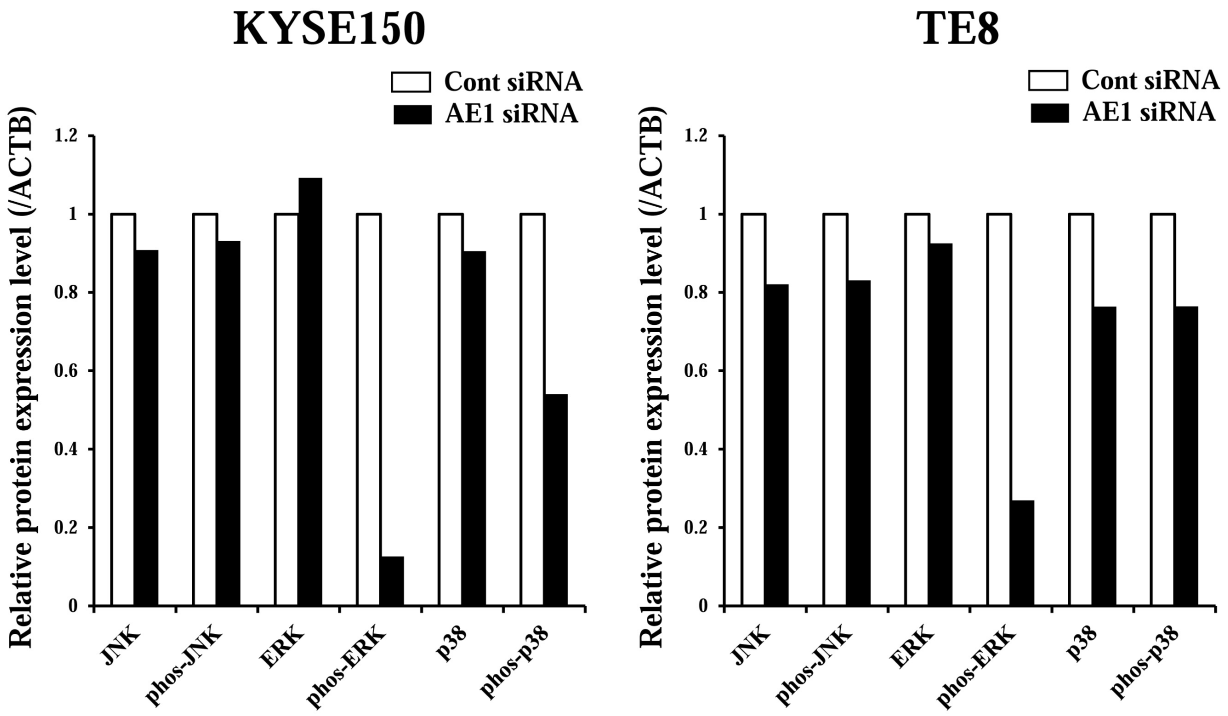

Supplementary Figure 7: MAPKs pathways regulated by AE1 in ESCC cells. The down-regulation of AE1 decreased the phosphorylation levels and/or the total protein levels of JNK, ERK, and p38 in KYSE150 and TE8 cells. Band densities were quantified using the ImageJ software after being scanned from the film (relative to ACTB).

Supplementary Table 1: Five-year survival rate of patients with pT1 ESCC according to various clinicopathological parameters

Variable	pT1 (n=27)	
	5-year survival rate (%)	p value
Gender		
Male	90.2	0.2186
Female	66.67	
Age		
<65 years	93.33	0.5106
≥65 years	82.5	
Tumor length		
<50 mm	85.6	0.4611
≥50 mm	100	
Histological type		
Well/moderately differentiated SCC	88.44	0.7004
Poorly differentiated SCC	83.33	
Lymphatic invasion		
Negative	100	0.0703
Positive	75	
Venous invasion		
Negative	88.67	0.6825
Positive	85.71	
pN		
pN0	100	0.0459*
pN1-3	72.92	
AE1 staining score		
Low	93.33	0.5106
High	82.5	
AE1 distribution		
Focal	100	0.3317
Diffuse	84.01	

SCC: squamous cell carcinoma; pT: pathological T stage; pN: pathological N stage.
*p<0.05: Log-rank test.

Supplementary Table 2: Prognostic factors of pT2-3 esophageal cancer according to a multivariate analysis

Variables		Risk ratio	95% CI	*p*-Value
Gender	Female	Ref		
	Male	2.044	0.451-14.899	0.375
Histological type	Well/moderately differentiated SCC	Ref		
	Poorly differentiated SCC	1.160	0.320-4.015	0.815
Lymphatic invasion	Negative	Ref		
	Positive	2.772	0.868-9.516	0.085
Venous invasion	Negative	Ref		
	Positive	2.705	0.810-11.211	0.109
pN	pN0	Ref		
	pN1-3	2.193	0.617-9.779	0.233
AE1 staining score	High	Ref		
	Low	1.680	0.433-6.057	0.438
AE1 distribution	Focal	Ref		
	Diffuse	4.154	0.970-21.495	0.055

SCC: squamous cell carcinoma; pN: pathological N stage; Ref: referent.
*$p<0.05$: Cox's proportional hazards model; 95% CI: 95% confidence interval.

Supplementary Table 3: Correlation between the pattern of postoperative recurrence within 5 years and expression of AE1

Variable	Staining score		p value	Distribution		p value
	Low (n=28)	High (n=33)		Focal (n=22)	Diffuse (n=39)	
Hematogenous recurrence						
(−)	25	30	0.832	22	33	0.016*
(+)	3	3		0	6	
Lymphogenous recurrence						
(−)	18	26	0.208	18	26	0.205
(+)	10	7		4	13	
Postoperative adjuvant chemotherapy						
(−)	16	14	0.251	10	20	0.662
(+)	12	19		12	19	

*p<0.05: chi-squared test.
Postoperative adjuvant chemotherapy: 5-FU plus cisplatin [13].

Supplementary Table 4: Twenty genes displaying the greatest change in expression levels in AE1-depleted KYSE150 cells

See Supplementary File 1

Supplementary Table 5: Top biological functions and canonical pathways of AE1 according to an Ingenuity Pathway Analysis

Top Biological Functions

Diseases and Disorders

Name	p value	Number of Molecules
Respiratory Disease	5.96E-03 – 1.46E-08	309
Cancer	6.03E-03 – 5.85E-08	2826
Gastrointestinal Disease	5.33E-03 – 5.85E-08	2243
Organismal Injury and Abnormalities	6.03E-03 – 5.85E-08	2841
Connective Tissue Disorders	5.07E-03 – 2.12E-07	57

Molecular and Cellular Functions

Name	p value	Number of Molecules
Cellular Movement	5.82E-03 – 9.00E-07	482
Cell Death and Survival	5.12E-03 – 2.07E-06	746
Cell Cycle	6.00E-03 – 2.45E-05	357
Cellular Development	5.07E-03 – 5.71E-05	456
Cellular Growth and Proliferation	5.07E-03 – 5.71E-05	728

Top Canonical Pathways

Name	p value	Ratio
Molecular Mechanisms of Cancer	2.30E-04	102/359 (28.4 %)
Axonal Guidance Signaling	5.52E-04	116/426 (27.2 %)
Pyridoxal 5’-phosphate Salvage Pathway	1.06E-03	24/63 (38.1 %)
Germ Cell-Sertoli Cell Junction Signaling	1.48E-03	48/155 (31.0 %)
Heme Biosynthesis from Uroporphyrinogen-III I	1.80E-03	4/4 (100 %)

Supplementary Table 6: Top 50 up- or down-regulated cell proliferation, cell cycle, apoptosis, migration, or invasion-related genes with expression levels in KYSE150 cells that were changed by the depletion of AE1

See Supplementary File 1
