# Supplementary material for: Expression and role of anion exchanger 1 in esophageal squamous cell carcinoma
Source: Oncotarget. 2017 Jan 30;8(11):17921–35. doi: 10.18632/oncotarget.14900 (PMC5392297; doi:10.18632/oncotarget.14900)
Supplement: Supplementary file 2 [file oncotarget-08-17921-s002.doc]

**Supplementary Table 4. Twenty genes displaying the greatest change in expression levels in AE1-depleted KYSE150 cells.**

| Up-regulated Genes | | | |
| --- | --- | --- | --- |
| Gene Symbol | UniGeneID | Gene Name | Exp Fold Change |
| MAGEA11 | Hs.670252 | melanoma antigen family A11 | 38.677 |
| ENOX1 | Hs.128258 | ecto-NOX disulfide-thiol exchanger 1 | 32.518 |
| ANKRD6 | Hs.702213 | ankyrin repeat domain 6 | 32.012 |
| ZFP28 | Hs.14794 | ZFP28 zinc finger protein | 31.228 |
| PKHD1 | Hs.662050 | polycystic kidney and hepatic disease 1 (autosomal recessive) | 28.737 |
| FCRL5 | Hs.415950 | Fc receptor-like 5 | 28.337 |
| SLC12A7 | Hs.172613 | solute carrier family 12 (potassium/chloride transporter), member 7 | 26.566 |
| EXOC6B | Hs.303454 | exocyst complex component 6B | 26.347 |
| ANK2 | Hs.620557 | ankyrin 2, neuronal | 23.595 |
| ZNF585A | Hs.729172 | zinc finger protein 585A | 23.249 |
| OR51B6 | Hs.553730 | olfactory receptor, family 51, subfamily B, member 6 | 22.663 |
| ACTL7B | Hs.534390 | actin-like 7B | 22.574 |
| CLC | Hs.889 | Charcot-Leyden crystal galectin | 20.902 |
| CACNB2 | Hs.59093 | calcium channel, voltage-dependent, beta 2 subunit | 19.626 |
| TEX15 | Hs.458316 | testis expressed 15 | 18.782 |
| PEX5L | Hs.235331 | peroxisomal biogenesis factor 5-like | 18.729 |
| SNRPN | Hs.632166 | small nuclear ribonucleoprotein polypeptide N | 18.252 |
| KRTAP13-3 | Hs.553687 | keratin associated protein 13-3 | 15.705 |
| BNC2 | Hs.656581 | basonuclin 2 | 13.739 |
| S1PR1 | Hs.154210 | sphingosine-1-phosphate receptor 1 | 13.519 |
|  |  |  |  |
| Down-regulated Genes | | | |
| Gene Symbol | UniGeneID | Gene Name | Exp Fold Change |
| DHH | Hs.524382 | desert hedgehog | -247.87 |
| IL23A | Hs.382212 | interleukin 23, alpha subunit p19 | -203.51 |
| FXYD3 | Hs.301350 | FXYD domain containing ion transport regulator 3 | -135.28 |
| DNAJB3 | Hs.725533 | DnaJ (Hsp40) homolog, subfamily B, member 3 | -120.93 |
| TMEM255A | Hs.437563 | transmembrane protein 255A | -107.48 |
| FAM81B | Hs.276287 | family with sequence similarity 81, member B | -99.862 |
| TRANK1 | Hs.170999 | tetratricopeptide repeat and ankyrin repeat containing 1 | -81.009 |
| NMNAT2 | Hs.497123 | nicotinamide nucleotide adenylyltransferase 2 | -74.521 |
| SLC9C1 | Hs.680112 | solute carrier family 9, subfamily C (Na+-transporting carboxylic acid decarboxylase), member 1 | -61.962 |
| SMYD1 | Hs.516176 | SET and MYND domain containing 1 | -49.745 |
| ICOS | Hs.56247 | inducible T-cell co-stimulator | -48.92 |
| CAPN13 | Hs.660911 | calpain 13 | -46.408 |
| HRH4 | Hs.287388 | histamine receptor H4 | -45.486 |
| PLCZ1 | Hs.97542 | phospholipase C, zeta 1 | -42.783 |
| ADCY8 | Hs.591859 | adenylate cyclase 8 (brain) | -41.35 |
| MAGEE2 | Hs.356869 | melanoma antigen family E2 | -40.657 |
| GSTM3 | Hs.2006 | glutathione S-transferase mu 3 (brain) | -40.367 |
| INPP5D | Hs.601911 | inositol polyphosphate-5-phosphatase, 145kDa | -36.423 |
| KRT14 | Hs.654380 | keratin 14, type I | -35.893 |
| ADIPOQ | Hs.80485 | adiponectin, C1Q, and collagen domain containing | -35.533 |

**Supplementary Table 6. Top 50 up- or down-regulated cell proliferation, cell cycle, apoptosis, migration, or invasion-related genes with expression levels in KYSE150 cells that were changed by the depletion of AE1.**

| Up-regulated Genes | |  |  |  |  |  |
| --- | --- | --- | --- | --- | --- | --- |
| Symbol | UniGene ID | Exp Fold Change | Biological Functions | | | |
|  |  |  | cell growth and proliferation | cell cycle | apoptosis | migration and invasion |
| MAGEA11 | Hs.670252 | 38.677 | ● |  |  |  |
| ANKRD6 | Hs.702213 | 32.012 | ● |  |  |  |
| PKHD1 | Hs.662050 | 28.737 | ● | ● | ● | ● |
| ANK2 | Hs.620557 | 23.595 | ● |  |  |  |
| CLC | Hs.889 | 20.902 | ● |  |  |  |
| S1PR1 | Hs.154210 | 13.519 | ● | ● | ● | ● |
| FOXF1 | Hs.155591 | 12.33 | ● |  |  | ● |
| CDX4 | Hs.553488 | 12.08 | ● |  |  |  |
| ADAM21 | Hs.178748 | 11.765 |  |  |  | ● |
| CDX1 | Hs.1545 | 11.391 | ● | ● | ● | ● |
| CXCL13 | Hs.100431 | 11.088 | ● |  |  | ● |
| SLC6A4 | Hs.29792 | 10.949 | ● | ● |  |  |
| DHRS2 | Hs.272499 | 10.747 | ● | ● | ● | ● |
| VIP | Hs.53973 | 10.102 | ● | ● | ● | ● |
| GFRA1 | Hs.388347 | 10.073 | ● | ● | ● | ● |
| EGR2 | Hs.1395 | 10.064 | ● | ● | ● | ● |
| PCDHGC3 | Hs.368160 | 9.625 | ● | ● | ● | ● |
| MUC13 | Hs.5940 | 9.617 | ● |  |  |  |
| AQP7 | Hs.455323 | 9.303 | ● | ● | ● | ● |
| CALB1 | Hs.65425 | 8.838 | ● | ● | ● | ● |
| GPM6A | Hs.75819 | 8.681 | ● |  |  | ● |
| CEACAM6 | Hs.466814 | 8.357 | ● | ● | ● | ● |
| ANXA10 | Hs.188401 | 8.348 | ● |  |  |  |
| PRKD1 | Hs.508999 | 8.27 | ● | ● | ● | ● |
| TP53TG5 | Hs.710271 | 8.146 | ● |  |  |  |
| EGR4 | Hs.3052 | 8.004 | ● | ● | ● | ● |
| SPHKAP | Hs.436306 | 7.81 | ● |  |  |  |
| FCER1A | Hs.897 | 7.518 | ● | ● | ● | ● |
| ZBTB20 | Hs.655108 | 7.494 | ● |  |  |  |
| ZBTB20 | Hs.655108 | 7.494 |  | ● |  |  |
| GPC5 | Hs.655675 | 7.398 | ● |  |  |  |
| CLEC1B | Hs.409794 | 7.236 | ● |  |  | ● |
| PLAG1 | Hs.14968 | 7.148 | ● | ● |  |  |
| CNOT2 | Hs.133350 | 6.812 | ● | ● | ● | ● |
| NAV1 | Hs.585374 | 6.597 |  |  |  | ● |
| SSH2 | Hs.654754 | 6.591 |  | ● |  |  |
| BATF2 | Hs.124840 | 6.57 | ● |  |  |  |
| CLEC4A | Hs.504657 | 6.568 | ● |  |  |  |
| CCNG2 | Hs.740456 | 6.526 | ● | ● |  |  |
| PTPRZ1 | Hs.489824 | 6.449 | ● | ● | ● | ● |
| NAA16 | Hs.512914 | 6.407 | ● | ● | ● | ● |
| PCSK6 | Hs.498494 | 6.345 | ● |  |  | ● |
| CALCRL | Hs.470882 | 6.343 | ● | ● | ● | ● |
| HMGA2 | Hs.505924 | 6.191 | ● | ● | ● | ● |
| RABGEF1 | Hs.530053 | 5.982 | ● |  |  | ● |
| CHRM3 | Hs.7138 | 5.925 | ● | ● |  | ● |
| JMY | Hs.482605 | 5.873 | ● | ● | ● | ● |
| COL11A1 | Hs.523446 | 5.835 |  |  |  | ● |
| SIRT4 | Hs.50861 | 5.812 | ● |  |  |  |
| SLC46A1 | Hs.446689 | 5.788 | ● | ● | ● | ● |
|  |  |  |  |  |  |  |
| Down-regulated Genes | |  |  |  |  |  |
| Symbol | UniGene | Exp Fold Change | Biological Functions | | | |
|  |  |  | cell growth and proliferation | cell cycle | apoptosis | migration and invasion |
| DHH | Hs.524382 | -247.87 | ● |  |  |  |
| IL23A | Hs.382212 | -203.511 | ● |  |  | ● |
| SMYD1 | Hs.516176 | -49.745 | ● | ● | ● | ● |
| ICOS | Hs.56247 | -48.92 | ● | ● | ● | ● |
| CAPN13 | Hs.660911 | -46.408 | ● |  |  |  |
| HRH4 | Hs.287388 | -45.486 |  |  |  | ● |
| INPP5D | Hs.601911 | -36.423 | ● | ● | ● | ● |
| KRT14 | Hs.654380 | -35.893 | ● | ● | ● | ● |
| ADIPOQ | Hs.80485 | -35.533 | ● | ● | ● | ● |
| INSL4 | Hs.418506 | -31.892 | ● |  |  |  |
| MGLL | Hs.277035 | -28.947 | ● |  |  | ● |
| ULBP1 | Hs.653255 | -28.836 | ● |  |  |  |
| NPPB | Hs.219140 | -28.534 | ● | ● | ● | ● |
| SCN9A | Hs.439145 | -28.18 |  |  |  | ● |
| ARC | Hs.40888 | -26.383 | ● | ● | ● | ● |
| SOX5 | Hs.657542 | -26.06 | ● | ● | ● | ● |
| ROBO2 | Hs.13305 | -25.413 | ● |  |  | ● |
| GPSM1 | Hs.239370 | -25.13 |  |  |  | ● |
| HPGDS | Hs.128433 | -23.35 | ● | ● | ● | ● |
| PPBP | Hs.2164 | -23.342 | ● |  |  | ● |
| CCL3 | Hs.514107 | -23.223 | ● | ● | ● | ● |
| KRT17 | Hs.2785 | -22.926 | ● | ● | ● | ● |
| SHROOM2 | Hs.567236 | -22.807 | ● | ● | ● | ● |
| DCHS1 | Hs.199850 | -22.111 | ● |  |  |  |
| MMP24 | Hs.715494 | -21.688 | ● |  |  |  |
| MB | Hs.517586 | -21.611 | ● | ● | ● | ● |
| IL2 | Hs.89679 | -20.801 | ● | ● | ● | ● |
| GPLD1 | Hs.533291 | -20.547 | ● | ● | ● | ● |
| ACKR1 | Hs.153381 | -20.186 |  |  |  | ● |
| TPM3 | Hs.644306 | -18.937 | ● |  |  | ● |
| COPZ2 | Hs.408434 | -18.841 | ● | ● |  |  |
| MTNR1B | Hs.569039 | -18.779 | ● | ● | ● | ● |
| SPG20 | Hs.440414 | -18.764 |  | ● |  |  |
| CFB | Hs.69771 | -18.718 | ● |  |  | ● |
| CLCN4 | Hs.495674 | -18.403 |  |  |  | ● |
| MCAM | Hs.599039 | -18.121 | ● | ● | ● | ● |
| COL1A2 | Hs.489142 | -17.512 | ● |  |  |  |
| OBSL1 | Hs.526594 | -17.508 |  | ● |  |  |
| HAS2 | Hs.159226 | -17.037 | ● | ● | ● | ● |
| FGD3 | Hs.411081 | -16.444 | ● |  |  |  |
| EFHC1 | Hs.403171 | -15.978 | ● | ● | ● | ● |
| STARD9 | Hs.122061 | -15.926 | ● | ● | ● | ● |
| NT5E | Hs.153952 | -15.797 | ● | ● | ● | ● |
| SCUBE3 | Hs.12923 | -14.684 | ● |  |  | ● |
| NEDD4L | Hs.185677 | -14.476 | ● | ● |  |  |
| P2RY6 | Hs.16362 | -14.456 |  |  |  | ● |
| IRX6 | Hs.369907 | -14.109 | ● |  |  |  |
| SYNGR1 | Hs.216226 | -13.604 | ● |  |  |  |
| CRIP2 | Hs.534309 | -13.428 |  |  |  | ● |
| DMBT1 | Hs.279611 | -13.229 |  |  |  | ● |
